# Prevalence of unprotected sexual activity in the Brazilian population and associated factors: National Health Survey, 2019

**DOI:** 10.1590/S2237-96222022000200027

**Published:** 2022-10-03

**Authors:** Tiago Odilio de Souza, Zeno Carlos Tesser, Ana Luiza Curi Hallal, Rodrigo Otavio Moretti Pires, Andreia Morales Cascaes

**Affiliations:** 1 Universidade Federal de Santa Catarina, Curso de Graduação em Medicina, Florianópolis, SC, Brazil; 2 Universidade Federal de Santa Catarina, Programa de Pós-Graduação em Odontologia, Florianópolis, SC, Brazil; 3 Universidade Federal de Santa Catarina, Programa de Pós-Graduação em Saúde Coletiva, Florianópolis, SC, Brazil

**Keywords:** Sexually Transmitted Diseases, Condoms, Sexual Behavior, Socioeconomic Factors, Adult Health, Cross-Sectional Studies

## Abstract

**Objective:**

To estimate prevalence of unprotected sexual activity and associated factors in the Brazilian population.

**Methods:**

This was a cross-sectional study with 61,523 adults aged 18 years or older who took part in the 2019 National Health Survey. We estimated prevalence of unprotected sexual activity in the last year. We analyzed association of socioeconomic and demographic variables with the outcome using Poisson regression, estimating prevalence ratios (PR) and 95% confidence intervals (95%CI).

**Results:**

Prevalence of unprotected sexual activity was 76.9% (95%CI 76.3;77.6), being higher in all the country’s regions in comparison to the Northern region, as well as being higher among people living in rural areas (PR = 1.04; 95%CI 1.03;1.06), females (PR = 1.06; 95%CI 1.05;1.08), participants aged 60 years or older (PR = 1.33; 95%CI 1.27;1.38), married individuals (PR = 1.25; 95%CI 1.23;1.27) and those with less education (PR = 1.05; 95%CI 1.03;1.06).

**Conclusion:**

Strategies aimed at groups with higher prevalence of unprotected sexual activity are necessary.

**Study contributions**

**Main results**

Unprotected sexual activity was reported by 77% of Brazilians. Prevalence of unprotected sexual activity was higher among females, people living in rural areas, those who were married, with less education and older adults.

**Implications for services**

The results emphasize the need to increase access to and raise awareness about condom use in groups with higher prevalence of unprotected sexual activity.

**Perspectives**

We suggest that further research be done that includes information on sexual orientation and gender identity, and which assesses sexual risk behaviors more comprehensively.

## Introduction

Sexually transmitted infections (STIs) are a serious global public health problem and have high reporting and detection rates every year.^1^ ^-^ ^3^ In Brazil, there have been more than 1.5 billion diagnosed cases of acquired syphilis, viral hepatitis and acquired immunodeficiency syndrome (AIDS) since 2010,^4^ and unprotected sexual activity at last intercourse has been shown to be positively associated with the prevalence of these outcomes.^5^ ^,^ ^6^ In 2020, the Brazilian public administration spent almost BRL 2 billion on medicines for the treatment of STIs,^7^ representing a 16% increase compared to expenditure in the previous year. Condom use is the main and most effective means of preventing STIs,^4^ besides avoiding early or unwanted pregnancies.^5^

Several factors can influence unprotected sexual activity. Social inequalities have a direct influence, both on risky sexual behavior and also on access to health services and adequate information, with the poorer and less educated strata of society being at greater risk of unprotected sexual activity.^8^ ^,^ ^9^ Furthermore, the need for female empowerment, especially to negotiate male condom use with partners, mirrors the increased prevalence of unprotected sexual activity among women,^10^ ^,^ ^11^ especially among older people. 

Having knowledge of the factors associated with unprotected sexual activity is a relevant matter and has been the object of investigation of regional studies.^11^ ^,^ ^12^ The latest National Health Survey (*Pesquisa Nacional de Saúde* - PNS), a nationwide household-based survey conducted in Brazil in 2019, collected information on the sexual activity of Brazilians. However, as of the time of this publication, there were still no studies that have investigated factors associated with unprotected sexual activity in the Brazilian population using data obtained from the 2019 PNS. Investigating these issues based on national surveys allows us to gather and have fundamental elements for planning action policies and health services aimed at addressing the factors associated with unprotected sexual activity and, in this sense, contribute to the prevention of STIs. Moreover, investigations of this nature provide information for monitoring and enhancing interventions currently underway. 

The objective of this study was to investigate prevalence of unprotected sexual activity in Brazil and its Federative Units, as well as factors associated with health risk behavior.

## Methods

### Design

This was a cross-sectional study that analyzed data from the 2019 National Health Survey (*Pesquisa Nacional de Saúde* - PNS), coordinated by the Brazilian Institute of Geography and Statistics (IBGE) in partnership with the Ministry of Health, conducted between August 2019 and March 2020.^13^ We analyzed data on 61,523 Brazilians aged 18 years or older who answered the module on sexual activity, which is part of the block of individual questions contained in the PNS.

### Background

The 2019 PNS is a population-based survey, representative of the population residing in private households in Brazil, located in urban and rural areas, macro-regions of the country, Federative Units, state capitals, and metropolitan regions. To this end, the survey coordinators and interviewers underwent training at IBGE’s state-level units.^13^

### Participants

The survey included individuals aged 15 years or older, living in permanent private homes. It excluded those living in Indigenous groups, barracks, military bases, lodgings, camps, boats, penitentiaries, penal colonies, prisons, jails, long-stay institutions for the elderly, integrated care networks for children and adolescents, convents, hospitals, settlement project villages and quilombola groups.^13^

Sampling for the 2019 PNS adopted a three-stage conglomerate plan: (i) selection of IBGE census tracts, primary sampling units, based on probability proportional to size, defined by the number of permanent private households in them; (ii) selection of permanent households in the coverage area, designated by simple random sampling; and (iii) the residents of each household, aged 15 years or older, also selected by simple random sampling.^13^ The 2019 PNS questionnaire was divided into three parts: (i) questions about the household, to be answered by the head of the household, (ii) general questions, about all residents of the household (e.g.: level of education, occupation, income, physical and/or intellectual disability, health insurance coverage, access to and use of health services), answered by a household member aged 18 years or older, and (iii) individual questions to be answered by a household resident aged 15 years or older.^13^ The module on sexual activity, the subject of this article, was part of the latter block of individual questions; however, it was answered only by individuals aged 18 years or older.^13^

**Figure 1:**
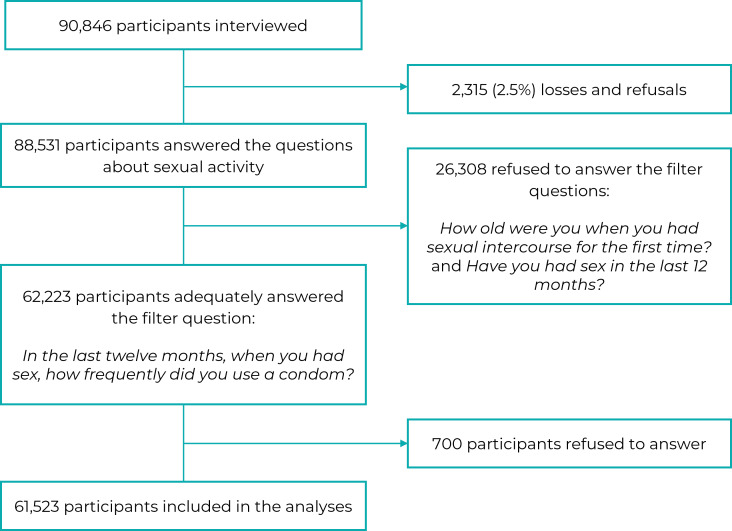
Sample selection process

The 2019 PNS randomly selected 94,114 Brazilians, 90,846 of whom were interviewed.^13^ Of these, 88,531 were household residents aged 18 years or older and therefore could have answered the questions about sexual activity. A filter question about having had sexual intercourse in the last 12 months was asked to estimate condom use and determine unprotected sexual activity. Of the participants who answered this question (n = 62,223), 61,523 provided complete information on the outcome (Figure 1). 

### Variables

The outcome variable was defined as "unprotected sexual activity", measured originally measured by asking the following question, *In the last twelve months, when you had sex, how frequently did you use a condom?*, the answers for which were: 1) Always; 2) Sometimes; 3) Not at all; 4) Refused to answer. For the purpose of the analysis performed in our study, the answer "Always" was placed in the "No (protected sexual activity)" category, while the answers "Sometimes" and "Not at all" were placed in the "Yes (unprotected sexual activity)" category. The "Refused to answer" category was classified as "missing" information.

The independent variables analyzed were:

- Demographic characteristics
  - Brazilian macro-regions (North; Northeast; Southeast; South; Midwest).
  - Zone of residence (urban; rural).
  - Age group (in completed years: 18-24; 25-39; 40-59; 60 and over).
  - Sex (male; female).
  - Race/skin color (White; Black; Indigenous; mixed race; Asian).
  - Marital status (single/widowed/divorced; married).
- Socioeconomic characteristics
  - *Per capita* household income (in quintiles).
  - Schooling (in years of study: up to 8; more than 8).

### Data source and measurement

The 2019 PNS database is a public domain database and can be accessed on IBGE’s website: https://www.ibge.gov.br/estatisticas/downloads-estatisticas.html. The data were collected in the selected households by applying an electronic questionnaire on mobile data collection devices, with self-reported questions answered in face-to-face interviews.^13^

### Bias control

The random sampling procedures using staged conglomerates adopted by the 2019 PNS aimed to minimize selection biases. All those responsible for data collection and supervision and coordination of the survey were trained to conduct it, with the aim of avoiding information biases.^13^ Expansion factors and sample weights were applied in the data analysis, this being a procedure justified by the complex sample design and distinct probabilities of selection enabled by the PNS.^13^

### Study size

Proportion estimation was taken into consideration in order to calculate the 2019 PNS sample size, with the expected level of accuracy described in 95% confidence intervals (95%CI). The effect of the sampling design was also taken into consideration.^13^ Furthermore, the number of households in each primary sampling unit was selected and the sample size was divided according to population subgroups.^13^ The sample calculation also considered the proportion of households with people in the age group of interest.^3^

### Statistical methods

The data were analyzed using Stata version 14.2 (StataCorp LP, College Station, United States), using the survey command, which considers stratification and conglomeration effects in the estimation of indicators and their measures of accuracy. We estimated the prevalence of unprotected sexual activity and respective 95%CI, both overall and also according to socioeconomic and demographic variables, both for Brazil as a whole and separately for its Federative Units. Poisson regression - crude and adjusted - was used to estimate the prevalence ratios (PRs) and the 95%CI for association between unprotected sexual activity and the other variables. All independent variables were considered theoretically important for model fit and were therefore included in the final adjusted model. The Wald test using 5% significance identified the variables associated with unprotected sexual activity, after adjusted analysis.

### Ethical aspects

The 2019 National Health Survey project was submitted to the National Health Council’s National Research Ethics Committee under Certificate of Submission for Ethical Appraisal No. 11713319.7.0000.0008, and was approved as per Opinion No. 3.529.376, issued on August 23, 2019.^13^ The PNS ensured the confidentiality of the information and personal data collected, and the consent of all respondents.^13^

## Results

Most participants lived in the Southeast region of Brazil (43.2%; 95%CI 42.2;44.2) and in urban areas (86.2%; 95%CI 85.8;86.7), more than half of the sample (52.2%; 95%CI 51.6;52.9) was male, and 38.2% (95%CI 37.5;38.9) were between 40 and 59 years old. The sample was composed predominantly of mixed race (44.2%; 95%CI 43.5;45.0) and White (43.0%; 95%CI 42.2;43.9) individuals, and 36.2% (95%CI 35.4;37.0) reported having up to eight years of schooling. Overall prevalence of unprotected sexual activity was 76.9% (95%CI 76.3;77.6). Proportionately, the Southern region had the highest prevalence of unprotected sexual intercourse (79.4%; 95%CI 78.1;80.7), as did residents of rural areas (81.6%; 95%CI 80.4;82.6) and female participants (78.8%; 95%CI 77.9;79.6). Prevalence of unprotected sexual activity reached 88.3% (95%CI 87.1;89.4) among the elderly, aged 60 years and older, and 87.5% (95%CI 86.9;88.2) among married people (Table 1).

**Table 1:** Characteristics of the population (n = 61,523), frequency of unprotected sexual activity and 95% confidence interval, according to socioeconomic and demographic variables, Brazil, 2019

Variable	Sample		Unprotected sexual activity
	n	% (95%CI^a^)	% (95%CI^a^)
**Brazilian macro-region**			
North	12,458	8.0 (7.7;8.4)	71.6 (69.9;73.2)
Northeast	20,440	25.5 (24.9;26.2)	78.0 (77.1;78.8)
Southeast	13,179	43.2 (42.2;44.2)	76.3 (75.1;77.6)
South	8,032	15.3 (14.8;15.9)	79.4 (78.1;80.7)
Midwest	7,414	8.0 (7.7;8.3)	77.4 (75.7;79.1)
**Zone of residence**			
Urban	47,137	86.2 (85.8;86.7)	76.2 (75.5;76.9)
Rural	14,386	13.8 (13.3;14.2)	81.6 (80.4;82.6)
**Sex**			
Male	33,197	52.2 (51.6;52.9)	75.2 (74.4;76.1)
Female	28,326	47.8 (47.1;48.5)	78.8 (77.9;79.6)
**Age group (in years)**			
18-24	6,355	14.6 (13.9;15.2)	57.7 (55.4;59.9)
25-39	22,806	36.2 (35.5;36.8)	76.1 (75.0;77.1)
40-59	24,453	38.2 (37.5;38.9)	81.8 (80.9;82.7)
≥ 60	7,909	11.0 (10.6;11.4)	88.3 (87.1;89.4)
**Race/skin color**			
White	22,091	43.0 (42.2;43.9)	78.2 (77.2;79.1)
Black	6,948	11.5 (11.0;11.9)	74.5 (72.6;76.4)
Asian	449	0.8 (0.7;0.9)	73.8 (66.7;79.8)
Mixed race	31,562	44.2 (43.5;45.0)	76.5 (75.6;77.4)
Indigenous	466	0.5 (0.4;0.7)	69.1 (55.5;80.0)
**Marital status**			
Single/widowed/divorced	33,444	50.4 (49.6;51.2)	66.5 (65.5;67.5)
Married	28,079	49.6 (48.8;50.4)	87.5 (86.9;88.2)
**Schooling (in years of study)**			
More than 8	36,560	63.8 (63.0;64.6)	74.1 (73.3;75.0)
Up to 8	24,963	36.2 (35.4;37.0)	81.9 (81.0;82.7)
***Per capita* household income (in quintiles)**			
1 (poorest)	10,306	12.8 (12.4;13.3)	77.1 (75.7;78.5)
2	10,622	16.4 (15.9;17.0)	76.9 (75.3;78.4)
3	12,140	20.7 (20.0;21.3)	77.0 (75.6;78.4)
4	12,368	22.4 (21.8;23.0)	76.6 (75.2;78.0)
5 (richest)	16,074	27.7 (26.8;28.6)	77.1 (75.8;78.3)

a) 95%CI: 95% confidence interval.

The Federative Units with the highest prevalence of unprotected sexual intercourse among male participants were Espírito Santo (80.7%; 95%CI 77.8;83.4) and Paraná (79.8%; 95%CI 76.7;82.6), while prevalence of unprotected sex among female participants was higher in Paraíba (86.8%; 95%CI 84.2;89.0) and Paraná (82.8%; 95%CI 79.8;85.4) (Supplementary Table 1). As for age group, especially among participants aged 18 to 24 years, unprotected sexual activity was most reported by people living in Bahia (66.9%; 95%CI 58.0;74.8) and Espírito Santo (66.3%; 95%CI 58.1;73.6). Among the elderly (≥ 60 years), prevalence of unprotected sexual intercourse was higher in Rio Grande do Norte (94.1%; 95%CI 90.2;96.6) and Sergipe (93.4%; 95%CI 89.8;95.8) (Supplementary Table 2).

**Table 2:** Characteristics of the population (n = 61,523), unprotected sexual activity, crude and adjusted prevalence ratios and 95% confidence intervals, according to socioeconomic and demographic variables, Brazil, 2019

Variable	Crude PR^a^ (95%CI^b^)	p-value^c^	Adjusted PR^a^ (95%CI^b^)^d^	p-value^c^
**Brazilian macro-region**		< 0.001		< 0.001
North	1.00		1.00	
Northeast	1.08 (1.06;1.11)		1.07 (1.05;1.10)	
Southeast	1.06 (1.03;1.09)		1.04 (1.01;1.07)	
South	1.10 (1.07;1.14)		1.08 (1.05;1.12)	
Midwest	1.08 (1.04;1.11)		1.08 (1.05;1.11)	
**Zone of residence**		< 0.001		< 0.001
Urban	1.00		1.00	
Rural	1.07 (1.05;1.08)		1.04 (1.03;1.06)	
**Sex**		< 0.001		< 0.001
Male	1.00		1.00	
Female	1.04 (1.03;1.06)		1.06 (1.05;1.08)	
**Age group (in years)**		< 0.001		< 0.001
18-24	1.00		1.00	
25-39	1.31 (1.26;1.37)		1.22 (1.17;1.27)	
40-59	1.41 (1.36;1.47)		1.25 (1.20;1.31)	
≥ 60	1.52 (1.46;1.59)		1.33 (1.27;1.38)	
**Race/skin color**		< 0.001		0.352
White	1.00		1.00	
Black	0.95 (0.92;0.98)		0.98 (0.95;1.01)	
Asian	0.94 (0.86;1.03)		0.95 (0.88;1.03)	
Mixed race	0.97 (0.96;0.99)		1.00 (0.98;1.01)	
Indigenous	0.88 (0.73;1.05)		0.92 (0.80;1.06)	
**Marital status**		< 0.001		< 0.001
Single/widowed/divorced	1.00		1.00	
Married	1.31 (1.29;1.33)		1.25 (1.23;1.27)	
**Schooling (in years of study)**		< 0.001		< 0.001
More than 8	1.00		1.00	
Up to 8	1.10 (1.08;1.12)		1.05 (1.03;1.06)	
***Per capita* household income (in quintiles)**		0.009		0.070
1 (poorest)	1.00		1.00	
2	0.99 (0.97;1.02)		1.00 (0.97;1.03)	
3	0.99 (0.97;1.02)		0.98 (0.96;1.01)	
4	0.99 (0.96;1.01)		0.97 (0.94;0.99)	
5 (richest)	0.99 (0.97;1.02)		0.97 (0.94;1.00)	

a) 95%CI: 95% confidence interval.

After adjusted analysis, prevalence of unprotected sexual intercourse was higher in all major regions of the country in comparison to the Northern region. It was also higher among people living in rural areas (PR = 1.04; 95%CI 1.03;1.06), among females (PR = 1.06; 95%CI 1.05;1.08), elderly (≥ 60 years: PR = 1.33; 95%CI 1.27;1.38), married people (PR = 1.25; 95%CI 1.23;1.27), and among those with the lowest levels of education (PR = 1.05; 95%CI 1.03;1.06) (Table 2).

**Table 3:** Combined effect between marital status and age and unprotected sexual activity (n = 61,523), and crude and adjusted prevalence ratios and 95% confidence intervals, Brazil, 2019

Marital status and age (in years)	PR^a^ crude (95%CI^b^)	PR^a^ adjusted (95%CI^b^)^c^
Single/widowed/divorced and 18-24	1.00	1.00
Single/widowed/divorced and 25-39	1.27 (1.21;1.33)	1.26 (1.20;1.32)
Single/widowed/divorced and 40-59	1.31 (1.25;1.38)	1.29 (1.23;1.36)
Single/widowed/divorced and ≥ 60	1.36 (1.28;1.44)	1.35 (1.27;1.43)
Married and 18-24	1.48 (1.38;1.58)	1.46 (1.37;1.56)
Married and 25-39	1.55 (1.48;1.63)	1.55 (1.48;1.63)
Married and 40-59	1.61 (1.54;1.69)	1.60 (1.53;1.68)
Married and ≥ 60	1.71 (1.63;1.79)	1.70 (1.62;1.78)

a) PR: Prevalence ratio; b) 95%CI: 95% confidence interval; c) Interaction between marital status and age, adjusted by region, zone of residence, sex, race/skin color, schooling and *per capita* family income. Note: P-value of the interaction test in the crude and adjusted analyses < 0.001.

An adjusted linear effect of age on unprotected sexual activity was found between strata of the "marital status" variable: the largest effect was found for the married and elderly groups: PR = 1.70; 95%CI 1.62;1.78 (Table 3).

## Discussion

Almost 80% of participants in the 2019 PNS engaged in unprotected sex in sexual intercourse in the last twelve months. Socioeconomic and demographic inequalities were identified in the prevalence of unprotected sexual activity. The highest prevalence of unprotected sexual activity was found among females, people living in rural areas, married individuals, people with lower education and older people. The Northern region had the lowest prevalence of unprotected sexual intercourse. The Northeast, Southeast, South, and Midwest regions reported similar prevalence rates between each other.

Some limitations of this study should be mentioned. It is noteworthy that, given the fact that the study was part of the 2019 PNS module on sexual activity, many people did not answer the filter question and many others may have provided socially desirable answers, which may have resulted in the outcome of the study being underestimated. Another limiting factor was that some questions about risky sexual behaviors, such as involvement with multiple sexual partners, were not further explored. A suggestion for future research would be for surveys to include a question related to the number of sex partners, so that this variable can be associated with condom use and thus enable sexual risk behaviors to be analyzed better. 

People living in the Northern region had lower prevalence of unprotected sexual intercourse. This finding is consistent with those of other studies, such as (i) a cohort, study conducted with over 5,000 Brazilians between 16 and 65 years old, which examined condom use trends in the Brazilian population between 1998 and 2005,^14^ and (ii) a cross-sectional study with national sexual health indicators of over 100,000 adolescents in 2015.^5^ People living in the North of the country report high prevalence of multiple casual sex partners,^6^ this being a behavior that may be associated with higher condom use among young people. Another cross-sectional study on sexual behavior, also based on 2019 PNS data,^15^ showed that using health services to get condoms was higher in the Northern region. However, this association should be analyzed with caution, since more socioeconomically advantaged groups are less dependent on public health services for obtaining free condoms. It should also be noted that, in the national analysis of adolescent sexual health indicators conducted in 2015,^5^ the Northern region had the highest rates of early sexual initiation and teenage pregnancy. It is known that low socioeconomic status may be related to low sexual knowledge and unplanned pregnancy.^16^ ^,^ ^17^

Unprotected sex was more prevalent in rural residents, similar to results described in a 2016 cross-sectional study,^18^ conducted in Nigeria with more than 6,000 women of reproductive age, which concluded that rural women were more likely not to use condoms. Similarly, in Sub-Saharan Africa, demographic surveys of 99,000 young people aged 15-24 years between 2011 and 2016 in four regions,^19^ found that prevalence of unprotected sexual activity was higher among people living in rural areas. In Brazil, a household survey conducted in 2013^20^ reported that access to free condoms, among 15 to 64 years old sexually active individuals, was higher in urban areas, suggesting, therefore, that the rural population should also be guaranteed easy and confidential access to condoms.

Higher prevalence of unprotected sexual activity was found among female participants. The same pattern was found in the United States in a cross-sectional study of 24,000 adults aged 18-44 years between 2006-2010 and 2011-2013,^21^ when women had higher prevalence of unprotected sex among the adult population. In South Africa, analysis conducted in 2005 of 10,000 young adults aged 15 to 24 years,^22^ revealed that being female was also associated with low condom use. These findings can be explained by men’s control over issues involving sexual intercourse, often implying submissive behavior from women, who may even be reprimanded when they suggest condom use by their partners.^23^ In the United States, a cross-sectional survey of 12,000 women, conducted between 2006 and 2010,^24^ unveiled that the methods most used by women were contraceptive pills and female sterilization, which makes condom use redundant when people think that this method is only for avoiding pregnancy and not for preventing diseases.

Higher prevalence of unprotected sexual activity was found among older adults, this being a finding consistent with those of national and international studies. In 2011, among elderly people receiving care under the Brazilian Family Health Strategy in an urban region,^25^ only 17% of those who were sexually active used a protection method. Similarly, a survey conducted in the United States^24^ found patterns of change in contraceptive use between 1995 and 2010, and showed that as people age, they tend not to use condoms because they want to have children, or even use other methods if they are no longer interested in having children. This may also explain high prevalence of unprotected sexual intercourse among married individuals, found both in this study and in another study mentioned above.^22^ In João Pessoa, capital of the state of Paraíba, a household survey conducted in 2013 with 300 single and married sexually active women,^26^ indicated out that trusting their partners was one of the main reasons why condom use was avoided by these women. That study also found an increase in prevalence of unprotected sexual activity as aged increased, being higher among married and older adults. If on the one hand less frequent protection between married people may reflect partners trusting each other, on the other hand, it is possible that an increase in prevalence of unprotected sex among married people occurs with increasing age, which may justify the interaction found between marital status and age. Such factors increase the risk for STIs since there is no absolute guarantee of fidelity between married partners.

People with less education reported higher frequency of unprotected sexual activity in this study. In Latin America and the Caribbean, socioeconomic inequalities are directly related to information about STI transmission and condom use.^9^ In these regions of the Americas, between 2008 and 2018, people living in countries with higher socioeconomic status were more likely to use condoms,^9^ both at first and last sexual intercourse. Another study, conducted in Sub-Saharan Africa based on demographic surveys conducted between 2011 and 2018 with unmarried adolescents between 15 and 19 years old,^27^ suggests that low education is a factor positively associated with unprotected sex and risky sexual behavior, such as having multiple partners. These data are reproduced in our country, where, according to the national STI indicators,^4^ rates of these diseases are higher among people with low levels of education. These findings highlight the need to discuss the effects of social inequalities on knowledge about STIs and the use of prevention methods in sexual intercourse. 

No association was found between unprotected sexual activity and the respondents’ family income or race/skin color. The effect of income on the outcome analyzed may be related to programs that provide free access to condoms, widely spread throughout the country, which may have contributed to reducing the influence of income inequalities on the outcome. The result regarding race/skin color follows the trends of condom use in the Brazilian population, observed between 1998 and 2005,^14^ when no association was found between not using condoms and race/skin color. STI cases were more prevalent in Brazilians of Black and mixed race/skin color, according to the epidemiological indicators for Brazil for the period from 2010 to 2021.^4^ In the city of Salvador, capital of the state of Bahia, higher STI prevalence in adolescents who were also of Black and mixed race/skin color was identified between 2012 and 2017.^28^ These data may be a result of this part of the population having difficulty in accessing health services, which they have always suffered, made worse by the Brazilian social structure.^29^ ^,^ ^30^ In Bahia, a descriptive study carried out with White, Black and mixed race women aged 25 years or older who were respondents of the 2008 IBGE National Household Sample Survey,^30^ revealed that Black women account for a higher percentage of those facing poor levels of access to health services, making it evident that this group should be a priority target of prevention policies and health information campaigns.

The results presented emphasize the need for the implementation of public policies and action strategies by Primary Health Care professionals within the Brazilian National Health System, directed toward the most vulnerable groups identified in this study, regarding health information and education, seeking to achieve greater awareness among the Brazilian population and to promote expansion of and increased adherence to condom use.

## Supplementary Table 1

### Prevalence of unprotected sexual activity (n = 61,523) and 95% confidence intervals, by sex, in the Federative Units, Brazil, 2019

**Table:**

Federative Units	Prevalence of unprotected sexual activity	
	Male (95%CI^a^)	Female (95%CI^a^)
Acre	68.2 (64.2;71.9)	67.7 (63.1;72.0)
Amapá	66.5 (60.8;71.8)	64.3 (59.0;69.2)
Amazonas	60.8 (56.9;64.6)	67.3 (63.7;70.7)
Pará	71.4 (66.4;76.0)	76.9 (73.5;80.0)
Rondônia	73.1 (69.1;76.7)	78.2 (74.6;81.5)
Roraima	67.7 (64.1;71.2)	72.2 (67.5;76.5)
Tocantins	77.4 (73.1;81.3)	82.1 (77.4;86.0)
Alagoas	72.1 (67.5;76.2)	76.9 (72.7;80.6)
Bahia	77.7 (74.4;80.7)	79.9 (76.8;82.7)
Ceará	75.1 (71.7;78.3)	81.0 (78.0;83.6)
Maranhão	71.9 (69.0;74.7)	79.0 (76.4;81.4)
Paraíba	76.8 (73.5;79.8)	86.8 (84.2;89.0)
Pernambuco	77.0 (73.7;80.1)	81.5 (78.8;83.9)
Piauí	75.1 (71.5;78.4)	76.7 (72.6;80.4)
Rio Grande do Norte	74.5 (70.4;78.2)	78.7 (75.1;82.0)
Sergipe	77.0 (73.0;80.6)	80.7 (76.9;84.0)
Goiás	78.2 (73.6;82.3)	81.7 (78.2;84.8)
Mato Grosso	69.2 (64.2;73.8)	78.2 (74.4;81.6)
Mato Grosso do Sul	78.8 (75.5;81.8)	80.8 (77.5;83.7)
Distrito Federal	74.1 (69.6;78.1)	74.0 (69.0;78.3)
Espírito Santo	80.7 (77.8;83.4)	82.7 (79.2;85.7)
Minas Gerais	78.5 (75.8;80.9)	80.3 (77.3;83.0)
Rio de Janeiro	76.4 (73.6;79.0)	76.2 (73.0;79.1)
São Paulo	71.9 (69.1;74.5)	77.4 (73.0;79.1)
Paraná	79.8 (76.7;82.6)	82.8 (79.8;85.4)
Rio Grande do Sul	78.2 (75.3;80.8)	78.9 (75.6;81.9)
Santa Catarina	77.6 (74.3;80.5)	78.8 (75.5;81.7)

a) 95%CI: 95% confidence interval.

## Supplementary Table 2

### Prevalence of unprotected sexual activity (n = 61,523) and 95% confidence intervals, by age group, in the Federative Units, Brazil, 2019

**Table:**

Federative Units	Prevalence of unprotected sexual activity			
	18-24 years (95%CI^a^)	25-39 years (95%CI^a^)	40-50 years (95%CI^a^)	≥ 60 years (95%CI^a^)
Acre	54.1 (46.2;61.8)	66.9 (61.9;71.4)	72.4 (68.4;76.0)	85.0 (78.2;89.9)
Amapá	50.3 (41.8;58.8)	65.0 (58.6;70.9)	72.8 (66.3;78.4)	83.1 (75.3;88.8)
Amazonas	51.4 (44.9;57.8)	60.7 (57.2;64.2)	71.2 (67.2;75.0)	78.8 (68.9;86.3)
Pará	57.6 (50.1;64.8)	74.9 (70.7;78.6)	78.7 (74.5;82.4)	84.2 (76.5;89.7)
Rondônia	54.4 (45.0;63.5)	74.4 (69.2;79.0)	82.2 (78.9;85.1)	91.8 (86.2;95.3)
Roraima	57.5 (50.8;64.0)	68.8 (63.6;73.5)	76.0 (71.5;79.9)	82.1 (74.9;87.6)
Tocantins	62.8 (53.4;71.2)	80.0 (74.9;84.3)	83.8 (79.3; 87.5)	90.3 (84.5;94.0)
Alagoas	57.5 (48.5;65.9)	72.1 (67.0;76.6)	81.0 (76.3;84.9)	87.8 (80.3;92.7)
Bahia	66.9 (58.0;74.8)	77.1 (72.9;80.8)	83.3 (79.6;86.4)	84.8 (78.3;89.5)
Ceará	60.1 (52.2;67.4)	77.1 (73.9;80.0)	82.7 (79.4;85.5)	90.7 (86.3;93.8)
Maranhão	62.9 (57.2;68.3)	73.3 (70.2;76.2)	81.3 (78.2;84.0)	86.0 (81.1;89.7)
Paraíba	64.1 (54.9;72.3)	80.5 (76.6;83.8)	86.9 (84.0;89.3)	93.2 (88.2;96.2)
Pernambuco	60.0 (52.9;66.8)	77.4 (73.6;80.9)	84.9 (82.3;87.1)	93.2 (89.8;95.5)
Piauí	54.2 (43.8;64.2)	70.6 (66.4;74.4)	86.4 (82.7;89.4)	92.6 (87.2;95.8)
Rio Grande do Norte	50.1 (41.8;58.4)	75.6 (71.5;79.3)	83.3 (79.6;86.4)	94.1 (90.2;96.6)
Sergipe	63.7 (53.9;72.4)	75.5 (71.1;79.4)	84.5 (81.6;87.0)	93.4 (89.8;95.8)
Goiás	57.0 (44.0;69.1)	78.8 (74.1;82.9)	85.5 (82.8;87.9)	92.4 (88.1;95.2)
Mato Grosso	50.6 (40.3;60.9)	73.6 (68.8;77.8)	80.6 (77.3;83.5)	85.1 (76.1;91.1)
Mato Grosso do Sul	55.4 (47.0;63.5)	82.9 (79.6;85.8)	82.5 (79.2;85.4)	90.4 (87.0;92.9)
Distrito Federal	57.4 (44.1;60.9)	72.2 (67.7;76.2)	80.2 (76.1;83.8)	82.6 (72.1;89.6)
Espírito Santo	66.3 (58.1;73.6)	79.8 (75.8;83.2)	86.6 (83.5;89.2)	88.2 (80.9;93.0)
Minas Gerais	57.8 (49.8;65.3)	79.9 (76.5;83.0)	83.3 (80.2;86.0)	91.3 (88.0;93.7)
Rio de Janeiro	54.5 (46.9;61.8)	74.7 (71.4;77.8)	80.4 (77.5;83.0)	88.6 (85.7;90.9)
São Paulo	52.0 (45.0;59.0)	74.6 (71.0;77.9)	79.2 (76.3;81.8)	85.4 (81.3;88.7)
Paraná	63.1 (54.0;71.3)	81.2 (78.2;83.8)	85.6 (82.6;88.2)	91.5 (88.0;94.0)
Rio Grande do Sul	61.4 (53.1;69.1)	77.8 (74.2;81.0)	81.0 (78.2;83.6)	88.2 (85.0;90.8)
Santa Catarina	60.1 (52.2;67.6)	74.4 (70.5;77.9)	84.0 (81.1;86.5)	88.8 (83.9;92.4)

a) 95%CI: 95% confidence interval.
